# 1-Chloro­methyl­sulfinyl-2-nitro­benzene

**DOI:** 10.1107/S1600536812043553

**Published:** 2012-10-27

**Authors:** Sabrina Benmebarek, Mhamed Boudraa, Sofiane Bouacida, Jean-Claude Daran

**Affiliations:** a Unité de Recherche de Chimie de l’Environnement et Moléculaire, Structurale (CHEMS), Université Mentouri-Constantine, 25000 Algeria; b Département Sciences de la Matière, Faculté des Sciences Exactes et Sciences de la Nature et de la Vie, Université Oum El Bouaghi, Algeria; c Laboratoire de Chimie de Coordination, UPR CNRS 8241, 205 route de Narbonne, 31077 Toulouse Cedex, France

## Abstract

In the title compound, C_7_H_6_ClNO_3_S, the nitro group forms a dihedral angle of 2.7 (4)° with the benzene ring. The bond-angle sum at the S atom is 303.7°. In the crystal, mol­ecules are linked by weak C—H⋯O hydrogen bonds, forming layers lying parallel to (-101).

## Related literature

For the biological and pharmacological activity of sulfoxides, see, for example: Melzig *et al.* (2009 ▶); Huang *et al.* (2010 ▶). For related structures, see: Yan (2010 ▶); Kobayashi *et al.* (2003 ▶).
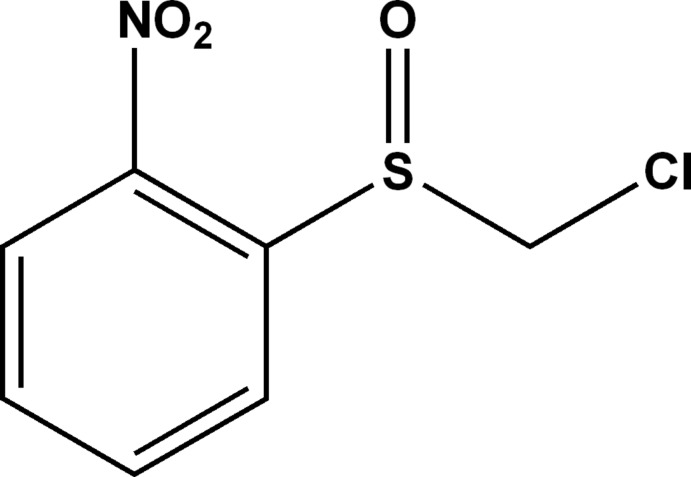


## Experimental

### 

#### Crystal data

C_7_H_6_ClNO_3_S
*M* *_r_* = 219.65
Monoclinic, 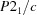

*a* = 12.2394 (5) Å
*b* = 5.5009 (2) Å
*c* = 14.5537 (11) Å
β = 116.631 (4)°
*V* = 875.92 (9) Å^3^
*Z* = 4
Mo *K*α radiation
μ = 0.65 mm^−1^
*T* = 180 K
0.23 × 0.20 × 0.18 mm

#### Data collection

Agilent Xcalibur (Eos, Gemini ultra) diffractometer
Absorption correction: multi-scan (*CrysAlis PRO*; Agilent, 2011 ▶) *T*_min_ = 0.900, *T*_max_ = 1.000
10209 measured reflections
2172 independent reflections
1799 reflections with *I* > 2σ(*I*)
*R*_int_ = 0.024

#### Refinement

*R*[*F* ^2^ > 2σ(*F* ^2^)] = 0.026
*wR*(*F* ^2^) = 0.071
*S* = 1.05
2172 reflections
118 parameters
H-atom parameters constrained
Δρ_max_ = 0.35 e Å^−3^
Δρ_min_ = −0.31 e Å^−3^

### 

Data collection: *CrysAlis PRO* (Agilent, 2011 ▶); cell refinement: *CrysAlis PRO*; data reduction: *CrysAlis PRO*; program(s) used to solve structure: *SIR2002* (Burla *et al.*, 2005 ▶); program(s) used to refine structure: *SHELXL97* (Sheldrick, 2008 ▶); molecular graphics: *ORTEP-3 for Windows* (Farrugia, 1997 ▶) and *DIAMOND* (Brandenburg & Berndt, 2001 ▶); software used to prepare material for publication: *WinGX* (Farrugia, 1999 ▶).

## Supplementary Material

Crystal structure: contains datablock(s) global, I. DOI: 10.1107/S1600536812043553/hb6974sup1.cif

Structure factors: contains datablock(s) I. DOI: 10.1107/S1600536812043553/hb6974Isup2.hkl

Supplementary material file. DOI: 10.1107/S1600536812043553/hb6974Isup3.cml

Additional supplementary materials:  crystallographic information; 3D view; checkCIF report

## Figures and Tables

**Table 1:** Hydrogen-bond geometry (Å, °)

*D*—H⋯*A*	*D*—H	H⋯*A*	*D*⋯*A*	*D*—H⋯*A*
C4—H4⋯O12^i^	0.95	2.44	3.384 (2)	173
C7—H7*A*⋯O1^ii^	0.99	2.36	3.2478 (18)	149
C7—H7*B*⋯O1^iii^	0.99	2.50	3.332 (2)	142

Symmetry codes: (i) 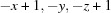; (ii) 
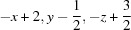
; (iii) 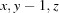.

**Fig. 1.**
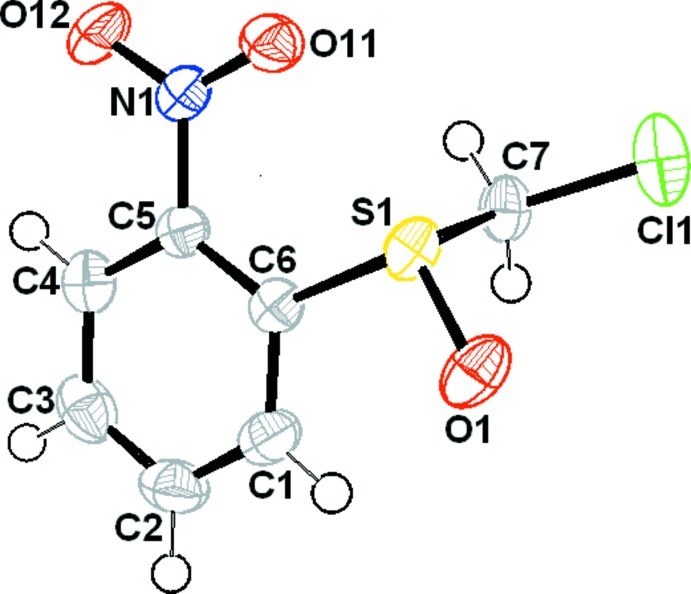
Drawing of asymetric unit of, (I), with displacement ellipsoids drawn at the 50% probability level.

**Fig. 2.**
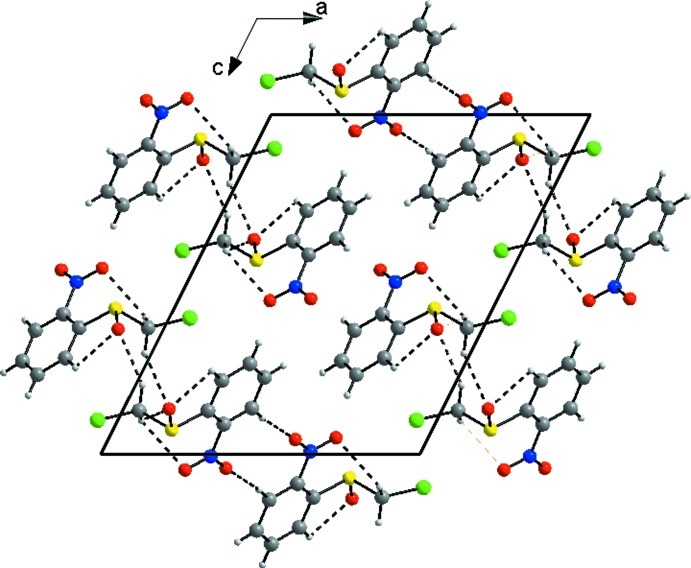
Diagram packing of (I) viwed via b axis showing alterning layers parallel to (-101) planes.

## supplementary crystallographic information

### Comment

The use of sulfoxides as pharmaceutical has shown promise in recent years (e.g. Melzig *et al.*, 2009 and Huang *et al.* 2010). As part of our ongoing studies on the synthesis, structures and biological activity of organometallic sulfanilamide complexes we have synthesized and determined the crystal structure of the title compound (I). The molecular geometry and the atom-numbering scheme are shown in Fig 1. In the crystal structure of the title compound, there are two pairs of molecules enantiomers in the unit cell. In each molecule, the nitro group forms a dihedral angle of 2.7 (4)° with the phenyl ring very different to that found in 2-(methylsulfinyl)benzamide (25.6°) (Yan, 2010) and in benzamide (26.31°) (Kobayashi *et al.*, 2003). The crystal packing is stabilized by weak C—H···O hydrogen bonds (Fig. 2) forming non-interacting layers parallel to (-101) planes.

### Experimental

*O*-chloronitrobenzene (1.60 g, 10 mmol) and thioacetic acid (0.80 g, 10 mmol) were dissolved in 75 ml aqua ethanol solution (25 ml water + 50 ml ethanol) and refluxed for 3 h under continuous stirring. Then the obtained product was evaporated at room temperature to dryness. The residue was diluted in 50 ml pure ethanol. After few days, orange bocks were recovered, as the solvent slowly evaporated.

### Refinement

All non-H atoms were refined with anisotropic atomic displacement parameters. Approximate positions for all H atoms were first obtained from the difference electron density map. However, the H atoms were situated into idealized positions and the H-atoms have been refined within the riding atom approximation. The applied constraints were as follow: C_aryl_—H_aryl_ = 0.95 Å and C_methylene_—H_methylene_ = 0.99 Å. *U*_iso_(H_aryl_/_methylene_) = 1.2*U*_eq_(C_aryl_/C_methylene_).

### Crystal data

**Table:**

C_7_H_6_ClNO_3_S	*F*(000) = 448
*M**_r_* = 219.65	*D*_x_ = 1.666 Mg m^−^^3^
Monoclinic, *P*2_1_/*c*	Mo *K*α radiation, λ = 0.71073 Å
Hall symbol: -P 2ybc	Cell parameters from 6334 reflections
*a* = 12.2394 (5) Å	θ = 3.3–29.2°
*b* = 5.5009 (2) Å	µ = 0.65 mm^−^^1^
*c* = 14.5537 (11) Å	*T* = 180 K
β = 116.631 (4)°	Block, orange
*V* = 875.92 (9) Å^3^	0.23 × 0.20 × 0.18 mm
*Z* = 4	

### Data collection

**Table:**

Agilent Xcalibur (Eos, Gemini ultra) diffractometer	2172 independent reflections
Graphite monochromator	1799 reflections with *I* > 2σ(*I*)
Detector resolution: 16.1978 pixels mm^-1^	*R*_int_ = 0.024
ω scans	θ_max_ = 29.2°, θ_min_ = 3.7°
Absorption correction: multi-scan (*CrysAlis PRO*; Agilent, 2011)	*h* = −16→15
*T*_min_ = 0.900, *T*_max_ = 1.000	*k* = −7→7
10209 measured reflections	*l* = −19→19

### Refinement

**Table:**

Refinement on *F*^2^	Primary atom site location: structure-invariant direct methods
Least-squares matrix: full	Secondary atom site location: difference Fourier map
*R*[*F*^2^ > 2σ(*F*^2^)] = 0.026	Hydrogen site location: inferred from neighbouring sites
*wR*(*F*^2^) = 0.071	H-atom parameters constrained
*S* = 1.05	*w* = 1/[σ^2^(*F*_o_^2^) + (0.0401*P*)^2^ + 0.1537*P*] where *P* = (*F*_o_^2^ + 2*F*_c_^2^)/3
2172 reflections	(Δ/σ)_max_ = 0.002
118 parameters	Δρ_max_ = 0.35 e Å^−^^3^
0 restraints	Δρ_min_ = −0.31 e Å^−^^3^

### Special details

**Table:**

Experimental. Absorption correction: Empirical absorption correction using spherical harmonics, implemented in SCALE3 ABSPACK scaling algorithm. CrysAlisPro (Agilent, 2011)
Geometry. All e.s.d.'s (except the e.s.d. in the dihedral angle between two l.s. planes) are estimated using the full covariance matrix. The cell e.s.d.'s are taken into account individually in the estimation of e.s.d.'s in distances, angles and torsion angles; correlations between e.s.d.'s in cell parameters are only used when they are defined by crystal symmetry. An approximate (isotropic) treatment of cell e.s.d.'s is used for estimating e.s.d.'s involving l.s. planes.
Refinement. Refinement of *F*^2^ against ALL reflections. The weighted *R*-factor *wR* and goodness of fit *S* are based on *F*^2^, conventional *R*-factors *R* are based on *F*, with *F* set to zero for negative *F*^2^. The threshold expression of *F*^2^ > σ(*F*^2^) is used only for calculating *R*-factors(gt) *etc*. and is not relevant to the choice of reflections for refinement. *R*-factors based on *F*^2^ are statistically about twice as large as those based on *F*, and *R*- factors based on ALL data will be even larger.

### Fractional atomic coordinates and isotropic or equivalent isotropic displacement parameters (Å^2^)

**Table:**

	*x*	*y*	*z*	*U*_iso_*/*U*_eq_	
Cl1	1.06418 (4)	0.73513 (8)	0.59930 (3)	0.03956 (13)	
S1	0.81710 (3)	0.81595 (6)	0.57165 (3)	0.02049 (10)	
O1	0.85724 (9)	1.04534 (18)	0.63222 (8)	0.0286 (2)	
O11	0.71137 (9)	0.42553 (19)	0.45234 (7)	0.0271 (2)	
O12	0.58622 (10)	0.15605 (19)	0.45752 (8)	0.0312 (3)	
N1	0.65255 (10)	0.3330 (2)	0.49290 (9)	0.0204 (2)	
C1	0.73727 (13)	0.7493 (3)	0.71720 (11)	0.0256 (3)	
H1	0.7829	0.8934	0.7453	0.031*	
C2	0.67575 (14)	0.6385 (3)	0.76600 (11)	0.0298 (3)	
H2	0.6802	0.7065	0.8276	0.036*	
C3	0.60801 (13)	0.4300 (3)	0.72587 (11)	0.0290 (3)	
H3	0.5672	0.354	0.7604	0.035*	
C4	0.59970 (12)	0.3319 (3)	0.63530 (11)	0.0238 (3)	
H4	0.5518	0.1908	0.6063	0.029*	
C5	0.66235 (12)	0.4428 (2)	0.58775 (10)	0.0186 (3)	
C6	0.73266 (11)	0.6514 (2)	0.62775 (10)	0.0188 (3)	
C7	0.94884 (12)	0.6136 (3)	0.62770 (11)	0.0231 (3)	
H7A	0.9786	0.6027	0.703	0.028*	
H7B	0.926	0.4484	0.5982	0.028*	

### Atomic displacement parameters (Å^2^)

**Table:**

	*U*^11^	*U*^22^	*U*^33^	*U*^12^	*U*^13^	*U*^23^
Cl1	0.0267 (2)	0.0429 (3)	0.0519 (3)	−0.00401 (16)	0.02018 (18)	0.00852 (19)
S1	0.02044 (17)	0.01600 (17)	0.02053 (17)	−0.00089 (12)	0.00517 (13)	0.00213 (13)
O1	0.0302 (5)	0.0156 (5)	0.0326 (5)	−0.0035 (4)	0.0074 (5)	−0.0018 (4)
O11	0.0317 (5)	0.0286 (6)	0.0247 (5)	−0.0052 (4)	0.0161 (4)	−0.0025 (4)
O12	0.0313 (6)	0.0277 (6)	0.0311 (6)	−0.0120 (4)	0.0109 (5)	−0.0111 (5)
N1	0.0192 (5)	0.0183 (6)	0.0205 (5)	0.0001 (4)	0.0061 (5)	−0.0008 (4)
C1	0.0237 (7)	0.0241 (7)	0.0235 (7)	0.0014 (6)	0.0057 (6)	−0.0049 (6)
C2	0.0300 (8)	0.0382 (9)	0.0207 (7)	0.0071 (6)	0.0110 (6)	−0.0023 (6)
C3	0.0263 (7)	0.0370 (9)	0.0270 (7)	0.0044 (6)	0.0148 (6)	0.0079 (7)
C4	0.0210 (7)	0.0228 (7)	0.0271 (7)	0.0004 (5)	0.0103 (6)	0.0032 (6)
C5	0.0171 (6)	0.0184 (6)	0.0178 (6)	0.0033 (5)	0.0054 (5)	0.0006 (5)
C6	0.0177 (6)	0.0171 (6)	0.0192 (6)	0.0033 (5)	0.0061 (5)	0.0022 (5)
C7	0.0191 (6)	0.0206 (6)	0.0279 (7)	0.0001 (5)	0.0091 (5)	0.0043 (6)

### Geometric parameters (Å, º)

**Table:**

Cl1—C7	1.7703 (14)	C2—C3	1.382 (2)
S1—O1	1.4908 (10)	C2—H2	0.95
S1—C6	1.8196 (14)	C3—C4	1.385 (2)
S1—C7	1.8236 (14)	C3—H3	0.95
O11—N1	1.2276 (15)	C4—C5	1.3837 (19)
O12—N1	1.2234 (15)	C4—H4	0.95
N1—C5	1.4618 (17)	C5—C6	1.3953 (18)
C1—C2	1.386 (2)	C7—H7A	0.99
C1—C6	1.3865 (19)	C7—H7B	0.99
C1—H1	0.95		
			
O1—S1—C6	104.99 (6)	C5—C4—C3	118.91 (13)
O1—S1—C7	105.15 (6)	C5—C4—H4	120.5
C6—S1—C7	93.53 (6)	C3—C4—H4	120.5
O12—N1—O11	123.31 (12)	C4—C5—C6	122.02 (12)
O12—N1—C5	118.97 (11)	C4—C5—N1	117.44 (12)
O11—N1—C5	117.72 (11)	C6—C5—N1	120.53 (12)
C2—C1—C6	120.49 (14)	C1—C6—C5	117.97 (13)
C2—C1—H1	119.8	C1—C6—S1	115.84 (11)
C6—C1—H1	119.8	C5—C6—S1	126.16 (10)
C3—C2—C1	120.60 (13)	Cl1—C7—S1	107.62 (7)
C3—C2—H2	119.7	Cl1—C7—H7A	110.2
C1—C2—H2	119.7	S1—C7—H7A	110.2
C2—C3—C4	119.97 (13)	Cl1—C7—H7B	110.2
C2—C3—H3	120	S1—C7—H7B	110.2
C4—C3—H3	120	H7A—C7—H7B	108.5
			
C6—C1—C2—C3	−0.6 (2)	C4—C5—C6—C1	−0.9 (2)
C1—C2—C3—C4	−0.9 (2)	N1—C5—C6—C1	179.16 (12)
C2—C3—C4—C5	1.5 (2)	C4—C5—C6—S1	−179.03 (10)
C3—C4—C5—C6	−0.6 (2)	N1—C5—C6—S1	1.02 (18)
C3—C4—C5—N1	179.38 (12)	O1—S1—C6—C1	−8.09 (12)
O12—N1—C5—C4	3.01 (18)	C7—S1—C6—C1	98.62 (11)
O11—N1—C5—C4	−177.48 (12)	O1—S1—C6—C5	170.09 (11)
O12—N1—C5—C6	−177.04 (12)	C7—S1—C6—C5	−83.20 (12)
O11—N1—C5—C6	2.47 (18)	O1—S1—C7—Cl1	−67.86 (9)
C2—C1—C6—C5	1.4 (2)	C6—S1—C7—Cl1	−174.44 (8)
C2—C1—C6—S1	179.78 (11)		

### Hydrogen-bond geometry (Å, º)

**Table:**

*D*—H···*A*	*D*—H	H···*A*	*D*···*A*	*D*—H···*A*
C4—H4···O12^i^	0.95	2.44	3.384 (2)	173
C7—H7*A*···O1^ii^	0.99	2.36	3.2478 (18)	149
C7—H7*B*···O1^iii^	0.99	2.50	3.332 (2)	142

Symmetry codes: (i) −*x*+1, −*y*, −*z*+1; (ii) −*x*+2, *y*−1/2, −*z*+3/2; (iii) *x*, *y*−1, *z*.
